# Manipulating the visibility of barriers to improve spatial navigation efficiency and cognitive mapping

**DOI:** 10.1038/s41598-019-48098-0

**Published:** 2019-08-09

**Authors:** Qiliang He, Timothy P. McNamara, Thackery I. Brown

**Affiliations:** 1 School of Psychology, Georgia Institute of Technology, Atlanta, USA; 2 Department of Psychology, Vanderbilt University, Nashville, USA

**Keywords:** Human behaviour, Cognitive neuroscience

## Abstract

Previous studies from psychology, neuroscience and geography showed that environmental barriers fragment the representation of the environment, reduce spatial navigation efficiency, distort distance estimation and make spatial updating difficult. Despite these negative effects, limited research has examined how to overcome barriers and if individual differences mediate their causes and potential interventions. We hypothesize that the reduced visibility caused by barriers plays a major role in accumulating error in spatial updating and encoding spatial relationships. We tested this using virtual navigation to grant participants ‘X-ray’ vision during environment encoding (i.e., barriers become translucent) and quantifying cognitive mapping benefits of counteracting fragmented visibility. We found that compared to the participants trained with naturalistic environment visibility, participants trained in the translucent environment had better performance in wayfinding and pointing tasks, which are theorized to measure navigation efficiency and cognitive mapping. Interestingly, these benefits were only observed in participants with high self-report sense of direction. Together, our results provide important insight into (1) how perceptual barrier effects manifest, even when physical fragmentation of space is held constant, (2) establish a novel intervention that can improve spatial learning, and (3) provide evidence that individual differences modulate perceptual barrier effects and the efficacy of such interventions.

## Introduction

Spatial navigation is one of the most fundamental functions in our daily life. Whereas it is easy to travel unimpeded to various locations in an environment with an open field structure such as African grasslands, it can be very challenging to find destinations in urban environments^1^ which are compartmentalized by barriers (e.g., doors, walls and buildings), without step-wise directions. A substantial amount of research from psychology, neuroscience and geography suggest that environmental barriers fragment the representation of the space^2–5^, decrease spatial navigational efficiency^6,7^, increase spatial updating difficulties across boundaries^8,9^ and distort distance estimation and direction judgments^10^. For example, studies on rodent’s place cells have shown that when rats navigated in an environment that was compartmentalized by doorways, place fields showed a high degree of spatial repetition and this repetition did not diminish with extended experience^11^. Furthermore, remapping of place cells was found to be purely local but not global^11^. Studies on rodent’s grid cells have shown that the signature six-fold periodic firing patterns of grid cells was disrupted when rats navigated a hairpin maze environment which was compartmentalized by barriers^2^. Similarly, humans’ grid-like signals were disrupted in barrier environments compared to open fields^12^. Together, these results implied that barriers interfere with forming a unified neural representation of the environment.

Given the amount of research showing effects imposed by barriers in spatial learning and memory, it is surprising that limited research has examined how to overcome barrier effects for improving spatial learning and memory and how individual differences factor into barrier effects and the efficacy of interventions. The current project adopted an applied perspective on this issue. We targeted the visibility component of barriers and we designed a perceptual virtual navigation intervention to improve people’s spatial navigation efficiency and cognitive mapping. Given theorizing that good navigators may be those who use perceptual cues more flexibly^13^, a critical applied aim was also to examine whether barrier intervention effects were of similar magnitude across individuals.

We define barriers in the current project as entities that can cause disruptions of continuous movement. The presence of such barriers often reduces both visibility^14^ and affordance^15^. Our previous study investigated the impact of affordance in spatial learning by rendering the objects in the virtual environment either impenetrable or penetrable^6^. Participants in that study either navigated in an impenetrable environment, in which they had to go around the obstacles to find the destinations, or they navigated in a penetrable environment, in which they could go through the obstacles to find the destinations. Critically, the visibility between the impenetrable and penetrable environments was controlled, with the barriers appearing opaque in both environments. The results of that study suggested that manipulating barriers’ affordance could increase map-like spatial knowledge (straight-line relationships between locations) but not navigation efficiency (wayfinding route performance connecting locations).

**Figure 1:**
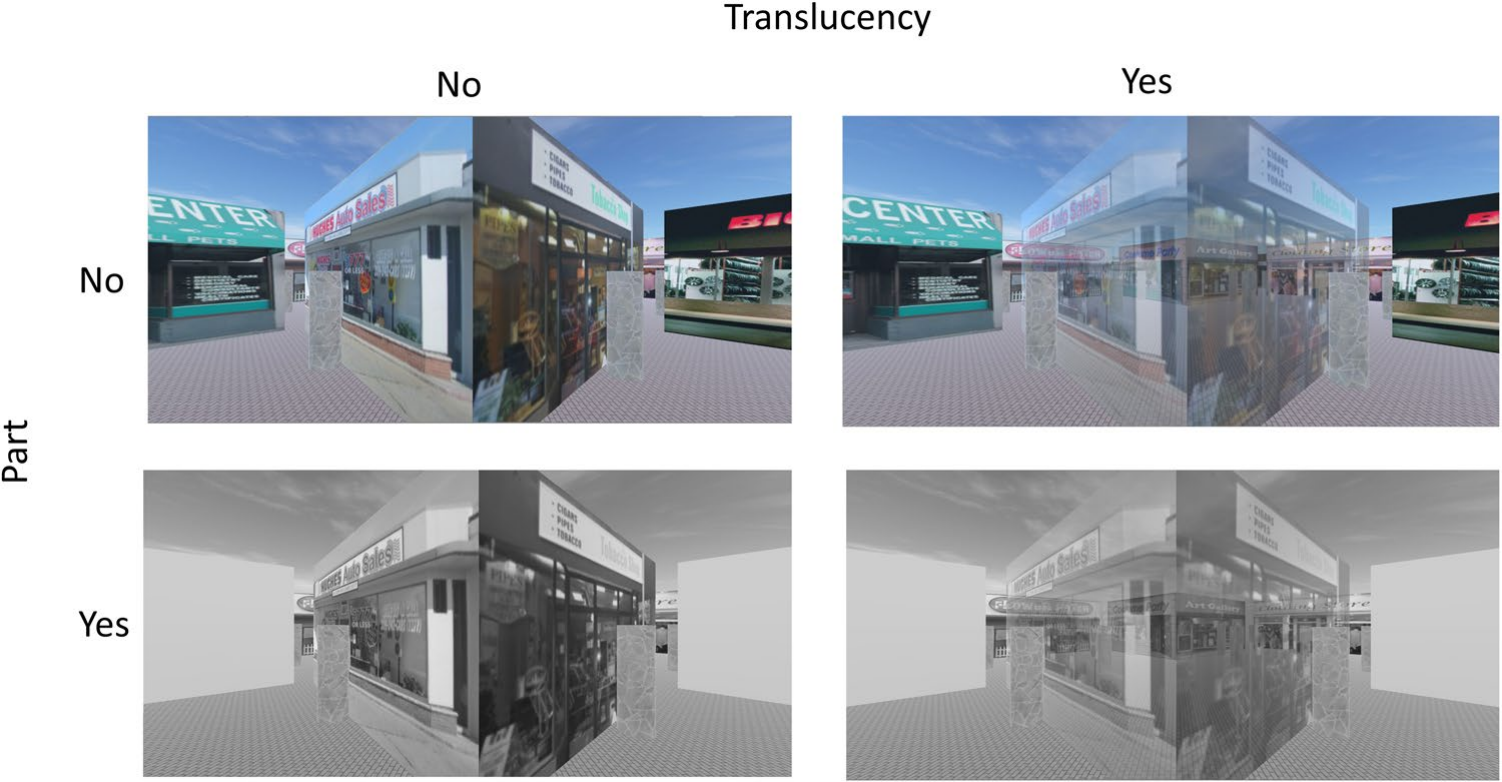
Illustrations of the translucent and the whole-part interventions in the training. Note that all participants, regardless of the conditions assigned, navigated in the whole, opaque environment in the testing phase. Upper left: whole-opaque condition, in which all buildings were presented and were opaque. Lower left: part-opaque condition, in which only a subset of buildings was textured at a time. All buildings were opaque. Upper right: whole-translucent condition, in which all buildings were presented but the building that the participant was looking at became translucent. Lower right: part-translucent condition, in which only a subset of buildings was textured at a time and the building that the participant was looking at (including an untextured one) became translucent. The bottom panels are greyed out to reflect the fact that the part-whole manipulation was not the main focus of the current project.

What role does barrier visibility play in cognitive map formation, and can wayfinding performance also be improved from virtual reality perceptual manipulations? From an applied perspective, it may be challenging for spatial learning that was based on structural affordance manipulations in virtual reality to transfer to the real/unmanipulated environment structure. Instead, in the current study we manipulated the visibility component of barriers and investigated how it impacted navigation efficiency and cognitive mapping. We reasoned that the reduced visibility caused by the presence of a barrier hinders direct perception of spatial relationships between locations. As a result, these spatial relationships have to be inferred, which is an error-prone process^16^. To counteract the reduced visibility, we rendered the barriers translucent (Fig. 1) in the virtual environment, while holding structural affordances constant, and quantified the impact of this manipulation on navigation efficiency and cognitive mapping, compared to an opaque environment. We rendered the objects translucent instead of transparent because translucency could allow participants relate landmarks near and far, and may increase learning-testing congruency^17^, which may increase spatial performance.

Weisberg and Newcombe^18^ previously demonstrated that spatial working memory capacity was correlated with cognitive mapping. From this, we conjectured (1) that reducing cognitive load (i.e., fewer stimuli competing for attention and working memory maintenance during path encoding) during spatial learning could also facilitate spatial knowledge acquisition, and (2) there was potential for barrier translucency to hinder, rather than enhance, spatial navigation by providing additional perceptual cues during learning that cannot be seen directly during subsequent retrieval under naturalistic visibility conditions. A long-established approach for simplifying learning of complex associations is to train people on one subset of task information at a time^19^(part-whole training), which could reduce cognitive and working memory load^20^. For this reason, we created a 2 × 2 design in which translucency and part-whole training were factorially combined.

A critical consideration, from both an applied and theoretical perspective, was the possibility that barrier effects (here, the efficacy of interventions that target them) may differ across individuals. It has been proposed by^13^ that good navigators are those who use landmarks and cues more flexibly. It is possible that good navigators could benefit more from the added cue visibility in our perceptual barrier intervention while physical affordances remain intact. To this end, we asked participants to report their sense of direction (SOD) and their spatial strategy preference^21^. SOD has been consistently shown to correlate with spatial ability^22,23^, and we categorized participants into high and low SOD groups to observe whether these two groups benefitted our intervention similarly.

## Methods

### Participants

Eighty-five participants from Georgia Institute of Technology and the Atlanta community participated in this experiment, either for course credits or monetary compensation. Participants spent 50–70 minutes completing the experiment. Five participants were unable to finish the experiment in 90 minutes and were therefore excluded from the study, leaving eighty participants in the data analysis (40 for translucent, 40 for opaque training conditions). Our target sample size was determined using a prospective power analysis on He *et al*.’s data^6^ upon which this study built. The power analysis showed that a sample size of 20 participants in each condition could achieve a power >0.80 for our measures of cognitive map memory. Recruitment continued until the two translucent conditions (part- and whole- training) and the two analogous opaque conditions had 20 participants each, with equal gender ratios (1:1; 10 females) in each condition.

Before the experiment, all participants completed the Questionnaire of Spatial Representation (QSR^21^) to evaluate SOD and spatial strategy preference. We calculated participants’ SOD scores as the sum of scores from items 1, 2, 3c, 8, 9 and 11 of the QSR (item 11 was reverse scored), because these questions were shown to be clustered into one factor, which was associated with sense of direction^21^ (these questionnaire items are detailed in Appendix).

All participants (age 18–23) gave written consent and informed consent was obtained from all participants. Participants were either paid or received course credits. The research was approved by the Institutional Review Board of Georgia Institute of Technology. All procedures were performed in accordance with the institutional guidelines.

### Materials and design

#### Independent variables

Using a between-subject design, the current project aimed to measure the impact of barriers on cognitive map formations from an intervention approach via two desktop VR manipulations: (1) we implemented a dynamic translucency algorithm in one group of participants. Here, proximal barriers would render translucent when in the center of the participant’s field of view (and opaque when distal or peripheral). (2) We implemented a part-whole manipulation in one group of participants. Here, we simplified the perceptual/cognitive load during encoding by rendering a subset of buildings texture-less in different encoding phases. Additionally, SOD was a key participant (quasi-independent) variable for our study. We detail the procedures for these independent variable manipulations below (see Procedure). The independent variables were factorially combined (i.e., opaque-whole, opaque-part, translucent-whole and translucent-part) and were implemented in the following environment:

#### Virtual environment

**Figure 2:**
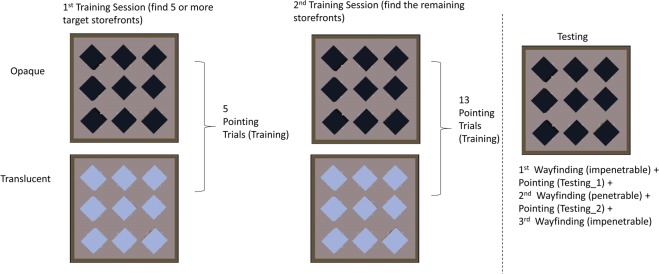
The training and testing procedures for opaque and translucent conditions. Dark blue diamond: opaque buildings. Light blue diamond: translucent buildings. Note that only one building could be translucent at a time. In the training phase, participants searched for the target storefronts in any order. In the wayfinding task of the testing phase, however, participants searched for the storefronts in a specific order (see main text). The first and third wayfinding tasks were performed in an impenetrable environment. An additional wayfinding task was performed in a penetrable environment to bridge our previous study^6^.

The virtual environment used in the current study was the same as the misaligned environment used in our previous study^6^ (Figs 1 and 2) and rendered in Unity3D (www.unity3d.com). The graphics were rendered for participants at a resolution of 1440 × 900 on a 19-inch monitor. The virtual environment consisted of a 50 m × 50 m (virtual “meters”) square enclosure and there were nine buildings inside the enclosure. Each building was 10 m (length) × 10 m (width) × 3 m (height) and had a unique storefront on each side. This resulted in 4 × 9 = 36 unique storefronts. One storefront from each building was selected as the potential target storefront, resulting in nine potential target locations. The same set of target storefronts was selected for all participants, based on^6^ (a random selection from the four possibilities on each building), to allow for clearer comparison with that work. A gray pole in front of each storefront served as the event-trigger object; during virtual navigation, participants were only considered to have reached a storefront when they collided with the pole in front of that storefront. The floor of the virtual environment was textured with a repeating tile pattern and the outer rectangular boundary wall was textured with a repeating brick pattern to provide simple optic flow. The sky was textured with a sky dome (Fig. 1).

#### Opaque and translucent environmental manipulation

Our findings revealed that the part-whole manipulation had no effect on any of the primary outcomes. We report these in the Results section (e.g., 3-way ANOVAs for translucency, part-whole, and SOD factors), but for simplicity we focus our manuscript on the primary translucent/opaque manipulation, collapsing across part- and whole- conditions. For readability, the following Methods sections describe the opaque (whole) and translucent (whole) manipulation (Fig. 1; Note that the part-whole condition procedures were identical between the opaque and translucent conditions, other than their specific perceptual manipulation, which is detailed in full in the Supplemental Materials).

In the opaque conditions (the combination of whole-opaque and part-opaque conditions), all buildings were presented naturalistically. In the translucent conditions (the combination of whole-translucent and part-translucent conditions), the building that participants were facing became translucent and it returned to be opaque when participants looked away (see video demo: https://osf.io/3bcxs). Note that the manipulation of translucency only occurred in the training phase, but not in the testing phase (see Procedure). We were interested in examining whether, compared to training in an opaque, naturalistic environment, training in a translucent environment could improve subsequent navigation efficiency and cognitive mapping.

#### Dependent variables

Navigation efficiency was quantified by excessive distance in the wayfinding task (see Procedure), which was defined as:

$$({\rm{actual}}\,{\rm{traversed}}\,{\rm{distance}}-{\rm{optimal}}\,{\rm{distance}})/{\rm{optimal}}\,{\rm{distance}}.$$

Excessive distance indicates how much further participants had traversed relative to the optimal distance. An excessive distance of 0 indicated perfect wayfinding performance (actual traversed distance equals optimal distance) and an index of 1 indicated the actual traversed distance was 100% longer than the optimal distance.

For the critical wayfinding task, optimal distance was computed as the distance of the shortest route between two locations, which was computed by DepthMapX^24^.

We used pointing error, an established measure of cognitive map formation^25–27^, to measure participants’ cognitive map accuracy. The pointing error was defined as the absolute (unsigned) angular difference between the actual angle and the response angle.

### Procedure

All experimental manipulations occurred during environment encoding (Training). Participants from all conditions received an identical testing phase in the naturalistic, opaque environment presentation (Fig. 1).

#### Training phase

After completing the QSR, participants practiced searching for various destinations in a distinct environment of the same size as that used in the experimental trials, but with only four buildings. A unique letter (A, B, C, etc.) was textured on each side of the buildings and participants were asked to find a specific letter. The practice trials ensured participants knew how to navigate in the virtual environment via keyboard and mouse. The training and testing procedures of these conditions are summarized in Fig. 2.

#### Learning in an opaque environment

Participants were instructed that the experiment included a training and a testing phase, and the environments between the training and testing phase were identical. Participants were encouraged to encode the spatial relationships between the target storefronts during the training phase so that they would find the testing phase easier.

**Figure 3:**
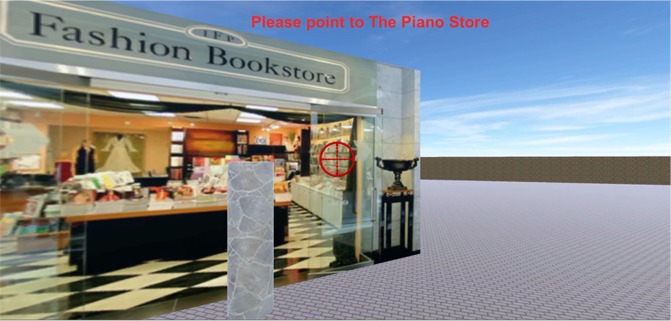
Illustration of a pointing task. Participants’ position was fixed at one of the target storefronts, and they were asked to use the crosshair to point to where the other target storefront was. All buildings except for the building at which participants were located were removed during the pointing task.

Participants were provided with a list of the nine target storefronts they needed to find throughout the experiment. Participants were told that they could find the target storefronts in any order they preferred in the training phase, and they would be asked to find these target storefronts again in the testing phase. There were two sessions in the training phase, each of which lasted at least 6 minutes to enforce a minimum exposure time exploring the environment. In the first training session, participants could freely explore the virtual environment, and were asked to find five (out of nine) of the target storefronts by navigating to the gray pole in front of it (Fig. 3). At the end of the first session, participants performed a pointing task of five pointing trials (Fig. 3). In each pointing trial, participants were teleported to one of the visited storefronts, and were asked to point to another visited storefront. During the pointing trial, participants could rotate their orientation but their position was fixed. In addition, all buildings except for the building at which they were located were removed (see video demo: https://osf.io/3bcxs). This procedure was used to (1) ensure that participants were informed of their current location after teleportation and (2) to encourage participants to rely on their cognitive map in the pointing task because other landmark cues were not available. After responding, participants were teleported to a different, visited storefront and repeated the process. The storefronts from which pointing judgments were made and the storefronts to which participants pointed were pseudorandomized to ensure that each storefront served both roles. Specifically, the pointing-from and pointing-to locations lists, each of which contained all nine target storefronts, were shuffled independently until there was no pair with the same pointing-from and pointing-to storefronts. This randomization process resulted in nine pointing trials and was repeated until eighteen trials were obtained for each block of the pointing task. The randomization of the pointing task was independent in each block and was independent of the randomization in the wayfinding task (see Testing phase). Pointing performance was measured by absolute angular error (see Dependent Variable section) and no feedback was given.

The second training session started after the pointing task was finished. The procedure was identical to the first training session and participants were simply asked to find the additional target storefronts which they did not find in the first session (participants were only required to find five storefronts in the first training phase). Participants were required to spend at least an additional six minutes in the second training phase (for a minimum of at least 12 minutes exploring the environment and found the 9 target storefronts across two sessions). If participants found all of the target storefronts in six minutes, then they were encouraged to keep exploring and learn the environment until time was up. Participants finished with thirteen pointing trials at the end of the second session.

#### Learning in a translucent environment

The procedures in this condition were identical to those in the opaque environment, except that we implemented the translucency manipulation described previously.

#### Testing phase

The testing phase took place in the naturalistic, opaque, whole version of the environment (Fig. 1). The testing phase consisted of wayfinding tasks and pointing tasks. In the wayfinding task, one of the target storefront names used in the training phase was presented on the screen and participants needed to find this storefront. When participants reached this storefront, the name of the next target storefront appeared on the screen and participants started the next wayfinding trial from the previous location. Participants had to find the target storefronts in a specific order in the testing phase, instead of in any order they preferred in the training phase. The order was randomized for each block and for each participant.

Each wayfinding task consisted of nine wayfinding trials, which contained all the nine target storefronts used in the training. The first and third wayfinding tasks were performed in an impenetrable environment. To bridge this study with He *et al*.^6^, the second wayfinding task was performed in a penetrable environment (visual presentation remained the same as in the impenetrable environment, but participants could navigate through the buildings to find the target; see video demo [https://osf.io/3bcxs] - penetrable). We had participants search for targets in impenetrable and penetrable environments because we found that wayfinding under different affordances measured different types of spatial knowledge^6^: Wayfinding in an impenetrable environment measures spatial knowledge that is more route oriented than the pointing task – enabling updating of heading as the trial unfolds and new cues become available around barriers. Wayfinding in a penetrable environment enables straight-line performance, where headings are not required to change in response to barriers. In the current study, we found that wayfinding performance in the penetrable environment, unlike impenetrable wayfinding, was equivalent under translucent and opaque training circumstances (see Supplement Fig. 1) and we focus in the main text on results from the naturalistic impenetrable test trials. The pointing task was the same as in the training phase, and each pointing task in the testing phase consisted of eighteen pointing trials. Compared to the wayfinding tasks, the pointing task is believed to better reflect participants’ cognitive map, as it requires judgments from a fixed position without the opportunity to update directional estimates as can be done when new cues emerge during the course of navigation.

The sequence of testing is summarized in Fig. 2 (pointing -> wayfinding -> pointing -> wayfinding -> pointing -> wayfinding, with the first pointing task in the training phase and the rest in the testing phase).

## Results

Overall self-report sense of direction (SOD) and spatial strategy preference (also calculated from the QSR) distributions were similar across conditions (Supplemental Table 1). Because we hypothesized that participants with different SOD could benefit from our interventions differently, we divided participants into high/low SOD groups by a median split of the SOD scores. Median split is a common statistical method in studies of individual differences in navigation^28–31^, and a recent review showed that it was appropriate when the independent variables are not correlated^32^, as in our study. There were eight participants with the median score, and it would be conceptually problematic to categorize participants with the same score to two different score groups. Therefore, participants with SOD score equal or larger than the median score were classified as high SOD participants and participants with SOD score lower than the median as low SOD participants. This resulted in forty-five participants in the high SOD group (22 opaque participants, 23 translucent) and thirty-five participants in the low SOD group (18 opaque participants, 17 translucent). All the patterns of results remained the same when we excluded the participants with the median score from the analyses.

For each spatial performance measure, we conducted a three-way ANOVA (SOD (high or low) X translucency X part-whole), and only conducted post-hoc comparisons on significant interactions. As results below show, the effect of the part-whole intervention was non-significant in almost all analyses. Therefore, we were able to combine the whole-opaque and part-opaque conditions as opaque conditions, and combine the whole-translucent and part-translucent conditions as translucent conditions for subsequent analyses.

### Wayfinding performance

#### First wayfinding test (impenetrable environment)

**Figure 4:**
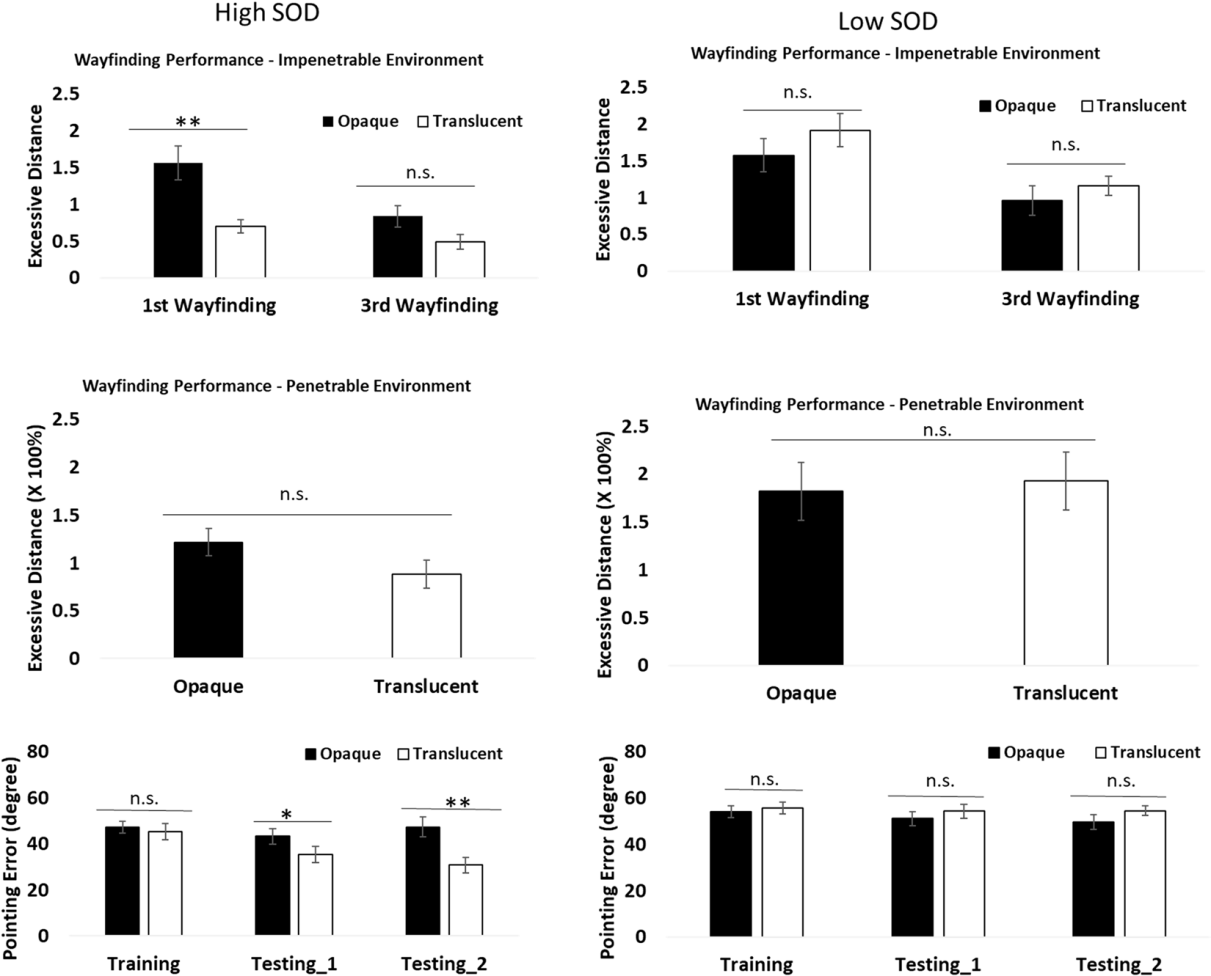
Wayfinding and pointing task performance for high and low SOD groups. Opaque conditions were a combination of whole-opaque and part-opaque conditions. Translucent conditions were a combination of whole-translucent and part-translucent conditions. Error bars are ±1 SEM estimated from data within conditions. n.s. non-significant, *p < 0.05, **p < 0.005.

Excessive distance on the first wayfinding task was analyzed in a 2 (SOD) × 2 (translucency) × 2 (part-whole) three-way ANOVA. The main effect of SOD was significant (*F*(1, 72) = 9.72, MSE = 0.78, *p* = 0.003, *η*^2^ = 0.12) as well as the interaction between SOD and translucency (*F*(1, 72) = 9.33, MSE = 0.78, *p* = 0.003, *η*^2^ = 0.12; Fig. 4, upper panels). No other main effects or interactions were significant (*F*s < 1.40, *p*s > 0.24). Post-hoc t-tests on the interaction showed that participants with high SOD in the combined translucent condition outperformed their own high SOD counterparts in the combined opaque condition (*t*(72) = −3.30, *p* = 0.001). This advantage was not significant, and was even reversed numerically, among the participants with low SOD (*t*(72) = 1.16, *p* = 0.25). These results reveal a benefit of translucency for navigation efficiency in the first wayfinding task for individuals with high SOD.

#### Third wayfinding test (impenetrable environment)

Excessive distance on the third wayfinding task was analyzed in a 2 (SOD) × 2 (translucency) × 2 (part-whole) three-way ANOVA. The main effect of SOD was significant (*F*(1, 72) = 7.19, MSE = 0.44, *p* = 0.009, *η*^2^ = 0.09). The interaction between SOD and translucency was marginally significant (*F*(1, 72) = 3.44, MSE = 0.44, *p* = 0.07, *η*^2^ = 0.05). No other main effects or interactions were significant (*F*s < 2.12, *p*s > 0.15). The main effect of SOD suggested that people with high SOD traversed shorter distance to destinations than did people with low SOD, although this was mainly driven by the translucent conditions (Fig. 4, upper panels).

### Pointing performance

#### Pointing task performance in training

Pointing error was analyzed in a 2 (SOD) × 2 (translucency) × 2 (part-whole) three-way ANOVA. The main effect of SOD was significant (*F*(1, 72) = 9.24, *MSE* = 162.17, *p* = 0.003, *η*^2^ = 0.11), as well as the interaction between SOD and part-whole (*F*(1, 72) = 4.30, MSE = 162.17, *p* = 0.042, *η*^2^ = 0.06). No other main effect or interaction was significant (*F*s < 2.47, *p*s > 0.12). Post-hoc t-tests on the interaction showed that participants with high SOD in the combined part condition outperformed their counterparts in the whole condition (*t*(72) = −2.60, *p* = 0.011), but this advantage was not significant among the participants with low SOD (*t*(72) = 0.46, *p* = 0.64).

#### Pointing task performance following the first wayfinding test

The pointing error of the first pointing task during testing was analyzed in a 2 (SOD) × 2 (translucency) × 2 (part-whole) three-way ANOVA. The main effect of SOD was significant (*F*(1, 72) = 12.59, *MSE* = 226.79, *p* = 0.001, *η*^2^ = 0.15), as well as the interaction between SOD and translucency (*F*(1, 72) = 4.24, MSE = 226.79, *p* = 0.043, *η*^2^ = 0.06; Fig. 4, lower panels). No other main effect or interaction was significant (*F*s < 1.77, *p*s > 0.19). Post-hoc t-tests on the interaction showed that participants with high SOD in the combined translucent condition had lower pointing errors than their counterparts in the combined opaque condition (*t*(72) = −2.62, *p* = 0.01), but this advantage was not significant among the participants with low SOD (*t*(72) = 0.43, *p* = 0.66).

#### Pointing task performance following the second wayfinding test

The pointing error of the third pointing task during testing was analyzed in a 2 (SOD) × 2 (translucency) × 2 (part-whole) three-way ANOVA. The main effect of SOD was significant (*F*(1, 72) = 14.09, *MSE* = 256.83, *p* < 0.001, *η*^2^ = 0.16), as well as the interaction between SOD and translucency (*F*(1, 72) = 8.04, MSE = 256.83, *p* = 0.006, *η*^2^ = 0.10; Fig. 4, lower panels). No other main effect or interaction was significant (*F*s < 3.18, *p*s > 0.08). Post-hoc t-tests on the interaction showed that participants with high SOD in the combined translucent condition had lower pointing errors than their counterparts in the combined opaque condition (*t*(72) = −4.64, *p* < 0.001), but this advantage was not significant among the participants with low SOD (*t*(72) = 0.65, *p* = 0.52).

Several studies^6,33^ have shown that the time course of cognitive map formation is slow. Therefore, we ran a 2 (SOD) × 2 (combined translucency vs opaque) × 3 (number of blocks; from training to testing_2) mixed ANOVA on the pointing task performance to examine whether the advantage of the translucent conditions became more evident as a function of time. The three-way interaction was significant (*F*(1, 152) = 3.41, MSE = 109.01, *p* = 0.036, *η*^2^ = 0.04; Fig. 4 lower panels), demonstrating that with more exposure in the environment, the advantage of participants with high SOD in the translucent condition over their same high SOD counterparts in the opaque condition became larger, but there was no such pattern among the participants with low SOD (Fig. 4, lower panels). On the other hand, the mixed ANOVA on wayfinding task performance did not generate a significant three-way interaction (*F*(1, 76) = 2.42, MSE = 76, *p* = 0.124, *η*^2^ = 0.03), probably due to the near ceiling performance in later wayfinding tasks.

All of the above analyses were based on categorical SOD, which is commonly used in previous studies^28–31^. We ran a moderation analysis to test whether continuous SOD was a moderator between the relationship between translucency and spatial learning. Translucency and continuous SOD were entered in the first step of the regression model and the interaction term (translucency X SOD) was entered in the second step. The interaction term explained a marginally significant increase in variance in the first wayfinding task (Δ*R*^2^ = 0.042, *F*(1, 76) = 3.83, *p* = 0.054) and a significant increase in variance in the final pointing task (second in the testing phase; Δ*R*^2^ = 0.062, *F*(1, 76) = 6.22, *p* = 0.015). Thus, SOD, regardless of whether we examined it as categorical or continuous predictor, played an important role in moderating the efficacy of our spatial intervention.

Overall, the results above showed

1. only participants with high SOD benefited from the translucency intervention, outperforming their high SOD counterparts in the first wayfinding task and the second pointing tasks. Such patterns were not observed among participants with low SOD.
2. participants with high SOD who were assigned to the translucent conditions outperformed participants with low SOD in both translucent and opaque conditions (wayfinding tasks: *t*s > 4.00, *p*s < 0.001; pointing tasks: *t*s > 4.18, *p*s < 0.001).

**Figure 5:**
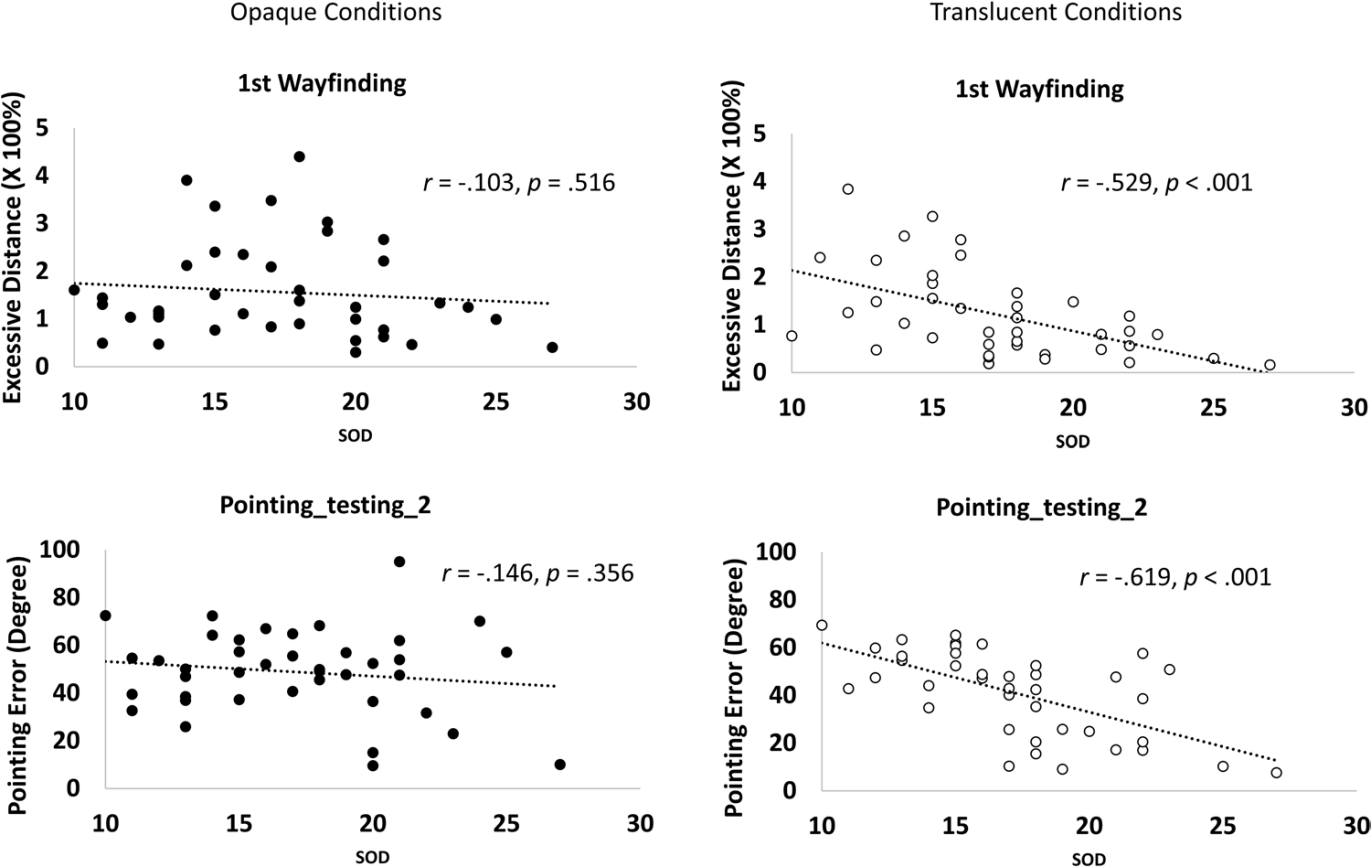
Correlations between SOD and spatial knowledge tasks performance.

We were struck by how strongly the SOD division was associated with whether individuals could utilize the translucency intervention to improve spatial performance. We further examined what SOD might reveal about an individual’s spatial learning process in addition to its face value (i.e., whether they use a cardinal, map-like representation to encode the space). In our prior observations with this general design^6^, we observed that SOD does not significantly predict or only had a weak correlation with cognitive map formation under naturalistic learning conditions (i.e., opaque conditions in the current study). First, we ran a correlation analysis of SOD and performance separately in the opaque conditions to see if we can replicate this pattern. As in our previous study, the correlation between SOD and the performance of the first wayfinding task was not significant in the opaque conditions (*r*(40) = −0.103, *p* = 0.516; Fig. 5), nor was the correlation between SOD and the performance in the final pointing task (*r*(40) = −0.146, *p* = 0.356; Fig. 5). On the other hand, in the translucent conditions, the correlation between SOD and the performance of the first wayfinding task was significant (*r*(40) = −0.529, *p* < 0.001, Fig. 5), as was the correlation between SOD and the performance of the final pointing task (*r*(40) = −0.619, *p* < 0.001, Fig. 5). A direct comparison demonstrated that these two correlations were significant higher in the translucent conditions than in the opaque conditions (*Z*s > 2.08, *p*s < 0.038).

Overall, the results from the correlation analysis showed that in the opaque environment, high SOD individuals struggled in wayfinding and pointing as much as their low SOD counterparts. This could be due to the convergence of effects imposed by barriers, the lack of global orientation cues, and the rotational symmetry of the enclosure. We expand on this topic in the Discussion section.

### Control analyses

**Figure 6:**
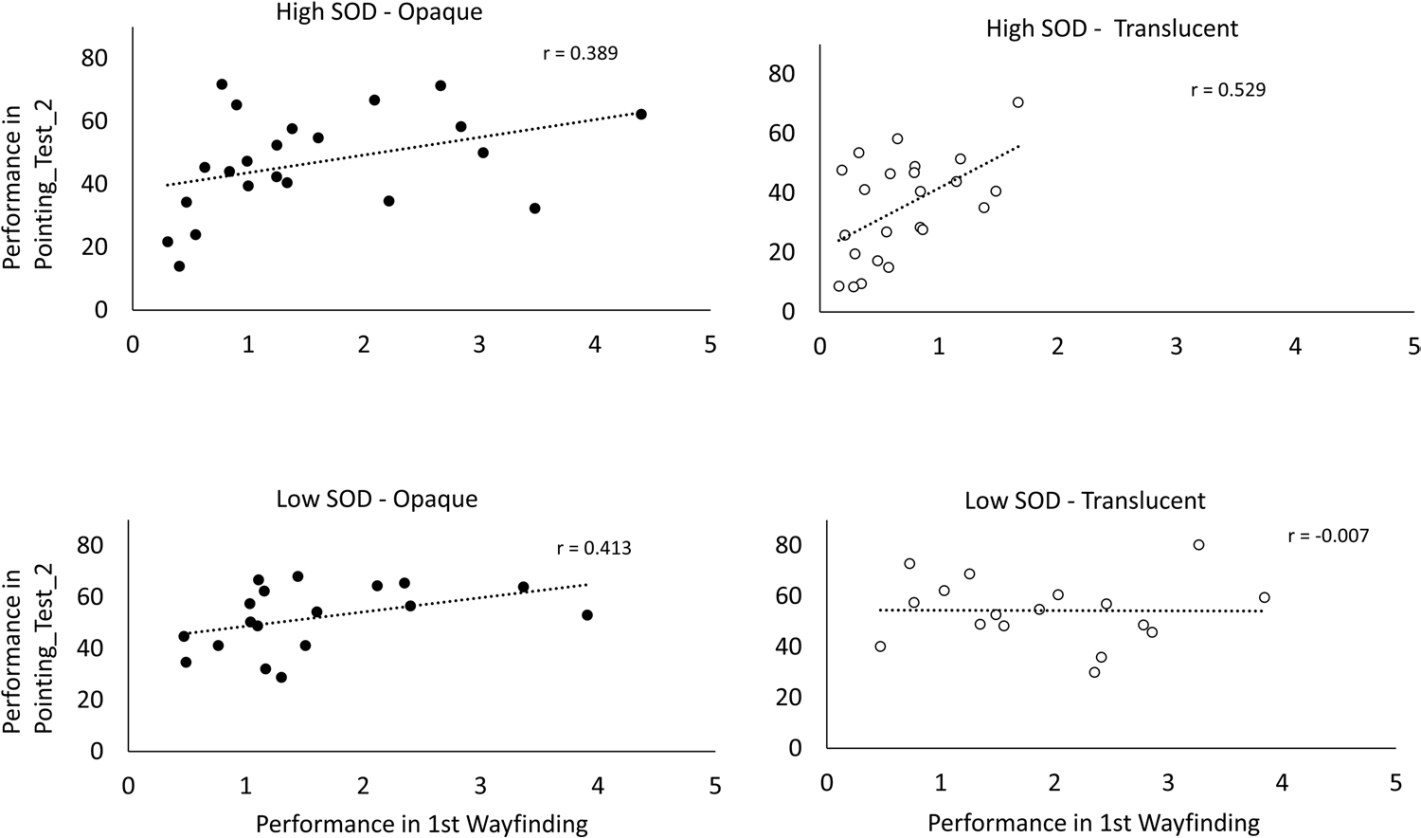
Correlations between wayfinding and pointing task performance for high and low SOD groups, separated by opaque and translucent conditions. Translucency appears to decouple pointing and wayfinding performance in the low SOD group, but strengthens the relationship in high SOD individuals.

We conducted control analyses to see if our results could be explained by variables other than our key manipulations. The analyses showed that the gender ratio, SOD scores, and, critically, training behavior (total traversed distance, total training time, amount of orientation changes [i.e., “searching” behavior] in the training phase) were not significantly different between the opaque and translucent conditions, and the significant differences in the wayfinding and pointing tasks between the opaque and translucent conditions remained significant when those factors were controlled for (Supplemental Table 2). In addition, the lack of a translucency effect in the participants with low SOD was not reflected in different training behaviors, as these indices (training time, distance and orientation change [amount of scanning]) were not significantly different from the participants with high SOD in the translucent conditions (*t*s < 1.39, *p*s > 0.17). Although the sample size was not intended for correlations at this level of group subdivisions, we also validated that the correlation between wayfinding and pointing task performance was above 0.38 in high SOD opaque and translucent groups, and in low SOD opaque condition. This replicates the typical relationship observed in the literature for these measures^6,34^. Interestingly, however, this correlation was effectively zero (r(17) = −0.007; Fig. 6) for the low SOD participants who were in the translucent conditions.

The differences between the opaque and translucent conditions were not driven by a specific *whole* or *part* condition (Supplement Fig. 2). Finally, SOD was correlated significantly with all dependent measures in the translucent conditions, but not with any measures in the opaque conditions (Supplemental Table 3). The patterns of correlations showed in Fig. 5 remained the same when gender effects were controlled for, and these patterns were not driven by a particular condition (Supplemental Table 4).

## Discussion

The current study aimed to investigate the way perceptual effects of barriers on spatial learning and memory, and whether we could overcome barrier effects using virtual reality interventions. We reasoned that the reduced visibility caused by barriers was one of the major factors, and we enhanced visibility by rendering the barriers translucent selectively during encoding to counteract this effect while maintaining natural spatial affordances. Our results showed that compared to the opaque, naturalistic environment, participants who were trained in the translucent environment had better performance in subsequent wayfinding and pointing tasks, which measure navigation efficiency and cognitive map formation. Interestingly, these improvements were only observed in participants with high SOD.

### Barriers and visibility

Despite the powerful influence of environmental barriers on the acquisition of spatial knowledge, limited research has investigated how barriers can impose these effects and even less research has studied how to overcome the presence of barriers for integrated map formation. As laid out in the Introduction, we consider that barriers have two major properties that may impose these effects: Reduced visibility and affordance. Our previous study^6^ manipulated the affordance of the barriers, and the current study manipulated the visibility of the barriers to assess how this impacted people’s spatial learning and memory.

We used wayfinding efficiency to represent navigation efficiency, and pointing error to represent cognitive map formation. For the wayfinding task, the benefits of translucency for high SOD participants appeared rather quickly and universally. This trend was reflected in non-significant differences between translucent and opaque learning scenarios in later tests, when wayfinding for opaque learning conditions also approached ceiling. For the pointing task, the benefit of translucency emerged gradually, facilitating cognitive map formation with more exposures to the environment. The gradual improvement may reflect the slow time course of integrating survey knowledge in a complex environment^6,25,33^. It is important to note that improved pointing performance for the translucency condition developed in the testing phase, while the environment was opaque (that is, without the translucency cues available). We speculate that although translucency did not benefit pointing task performance during initial training, it could help participants with high SOD to form a ‘spatial schema’ (a coarse cognitive map that could be reflected in the early wayfinding task benefit) in which new information can be incorporated into this schema and memory integration is facilitated^35^. Overall, our results show that the enhanced visibility could benefit participants with high SOD, enabling them to have better navigation efficiency and cognitive map. It suggests that at least for high SOD individuals, one way that barriers impose their effects on spatial learning and memory is by reducing availability of non-local cues that can facilitate cognitive map formation.

### Applied implications

Finding various destinations in a new environment can be challenging to many people, especially when the structure of the new environment is irregular and unpredictable^1,36^. Navigation aids, such as GPS, were designed to mitigate wayfinding challenges. However, a number of studies^37–39^ have shown that compared to non-GPS users, GPS users’ spatial representations of the environment are less accurate and their wayfinding performance is worse when GPS is not available. A recent study^40^ even suggest that prolonged use of GPS could impair the ability to learn new environments.

Unlike GPS, our data suggest that our translucency intervention in virtual navigation can enhance both wayfinding efficiency and cognitive map formation. One potential hurdle for applied settings is that to improve real world navigation with such an intervention, one has to create a virtual replica of the real environment. However, as mapping service providers such as Google Maps provide development tools to allows users to convert real world environments to virtual reality directly (https://cloud.google.com/maps-platform/gaming/?&sign=0), creating a virtual replica has become increasingly tractable. Not everyone could benefit from this intervention, but it was very effective for participants with high self-reported SOD. Critically, it is very quick and easy to measure SOD and to implement the translucency algorithm in virtual environments. As previous studies have shown that the transfer of training in virtual reality can be an effective substitute for training in real environments^41–43^, the benefits of our intervention could be three-fold: (1) We show that translucency training transfers to navigation of opaque environments. Therefore, once training is completed, there may be no need for physical or electronic navigation aids for superior performance in applied settings. As a result, trainees’ navigation performance need not be affected by issues such as GPS signal loss and device reliability. (2) Trainees can not only find their destination faster, but may have a greater ability to find detours or shortcuts enabled by their more accurate cognitive map. (3) Training in virtual reality is potentially much cheaper than on-site training, and can be used on a massive spatial scale in almost any environment. Taken together, our intervention could be of great benefit in applied settings where personnel are required to get to destinations in crowded and/or hazardous environments as quickly and as flexibly as possible, especially if there are particularly high risks or costs to learning a cognitive map or risking an uncertain detour “on the job” (e.g., ambulance drivers or soldiers deploying in unfamiliar settings).

### Individual differences and SOD

One of the consistent findings in the current study was that SOD played a profound role in predicting whether individuals can benefit from the translucency intervention. We hypothesized that high SOD participants may benefit more from the translucency intervention, but we did not expect that low SOD participants would not benefit at all. SOD measures individual’s self-report spatial ability, and has been shown to correlate with spatial updating and acquisition of spatial knowledge abilities^23^. Our correlation analysis (Fig. 5) implied that SOD could also measure individuals’ abilities (or tendencies) to utilize the environmental cues to form a cognitive map. Importantly, we show that in an environment which has few non-local location-informative cues (opaque conditions), SOD did not significantly predict spatial performance. Only in an environment where spatial cues beyond local physical space could be more directly observed (translucent conditions) did SOD significantly predict spatial performance. In other words, the predictive power of SOD not only depended on tasks^23^, but also on environmental visibility and/or cue availability. Our observation might help account for the wide range of correlations between SOD and spatial performance across studies (e.g., ranging from non-significant in^44^ to r = −0.18 in^26^ to r = −0.56 in^22^, despite similar task types), as our opaque and translucent conditions generated similar range of correlation coefficients (r = −0.146 and r = −0.619 for the last pointing task in the opaque and translucent conditions respectively).

Another interesting finding was the pattern of results for low SOD participants in the translucent conditions. Behavior during training in this group of participants did not differ from high SOD participants in the translucent conditions. As a group, high SOD group subsequent memory performance was no better than their low SOD counterparts in the opaque conditions. However, our control analyses (Fig. 6) revealed one interesting difference: low SOD wayfinding and pointing task performance was basically independent when these individuals learned under translucency. For other conditions, we replicated the typical positive correlation observed in other studies between these two measures^6,34^. This was not an anticipated outcome, but it suggests that translucency may not only benefit cognitive mapping in high SOD individuals, but also encourage divergent learning approaches in low SOD individuals. That is, some low SOD participants may be biased towards learning the route affordances connecting target storefronts whereas others may emphasize straight-line spatial relationships between locations that support pointing task performance but relate less-directly to *in situ* wayfinding.

Another possible reason of the lack of benefit among low SOD participants was that these participants might be less capable of orienting themselves using less informative spatial cues, such as the misaligned environmental barrier layout and the isometric square enclosure in the current study. As a result, low SOD participants may find it very difficult to incorporate the additional spatial information enabled by the translucency into their overall representation of the environment. In other words, being able to stay oriented could be important for our intervention effect. However, this and the aforementioned reason are highly speculative and future research are needed to test these hypotheses.

### Limitations and future directions

The current study opens several interesting lines of research. First, because SOD is so widely used in spatial navigation studies, it is very important that we establish when SOD can be a more or less direct predictor of performance. Our results suggest that SOD is a more powerful predictor when non-local cues are available in the environment that allow individuals’ to better relate locations of interest. Second, our results implied that the spatial learning strategy might be very heterogeneous within healthy young adults. Continued research on this topic may further shed light on the marked individual differences in spatial ability^13^.

Secondly, from the applied perspective, it is unknown whether our translucency intervention could enhance spatial learning in more complex or larger-scale environments. The current study was designed as a direct follow-up to our prior work^6^, and used a simple and tightly-controlled environment in which participants in the translucent conditions only needed to see through one barrier to see key landmarks. In real life, however, our current position and goal locations can often be separated by multiple layers of irregularly structured barriers, and we predict that our current translucency algorithm, which only enabled participants to see through one layer of barriers, would not improve spatial learning significantly in larger or more complex environments. Our lab is designing more adaptive translucency algorithms to enable participants to control how many layers of barriers they want to see through. We hypothesize that the adaptive translucency algorithm could facilitate spatial learning in more naturalistic, large scale and complex environments, at least for high SOD people.

Finally, another limitation of the current study is the lack of body-based cues during navigation, which is shown to facilitate spatial learning and updating^6,44–46^. Therefore, future research conducted in immersive virtual reality not only could provide more ecological validity, but also could investigate whether low SOD participants could benefit from the translucency intervention when they stay oriented in the environment better due to the body-based cues. The findings from such continued research could in turn shed light on important questions about what SOD measures besides one’s general spatial ability, and the mechanisms through which SOD impacts the efficacy of spatial intervention.

## Supplementary information

Supplementary Information

## Data Availability

The data that support the findings of this study and the analysis code are available from the corresponding authors upon request.
