# Supplementary material for: Manipulating the visibility of barriers to improve spatial navigation efficiency and cognitive mapping
Source: Sci Rep. 2019 Aug 9;9:11567. doi: 10.1038/s41598-019-48098-0 (PMC6688987; doi:10.1038/s41598-019-48098-0)
Supplement: Supplementary file 1 — Supplementary Information [file 41598_2019_48098_MOESM1_ESM.pdf]

Supplemental Material

Manipulating the visibility of barriers to improve spatial navigation efficiency and cognitive mapping

Qiliang He[1], Timothy P. McNamara[2], Thackery I. Brown[1*]

[1]School of Psychology, Georgia Institute of Technology, USA

[2]Department of Psychology, Vanderbilt University, USA

*Correspondence concerning this article should be addressed to Thackery I. Brown, School of Psychology, Georgia Institute of Technology, USA. Email: thackery.brown@psych.gatech.edu.

Procedure for part and whole conditions

As described in the main text, the procedure differences among all experimental conditions (whole_opaque, whole_translucent, part_opaque and part_translucent) were only in the training phase and the descriptions of the testing phase can be found in the main text. Therefore, we only describe training phase of the whole and part conditions here.

Whole conditions.

Participants were presented with a virtual town with all buildings fully textured. Participants were first provided a list of the names of the target storefronts (9) and five of which were marked with a check mark. Participants were told to find the storefronts with a check mark first, in any order, but we emphasized that all 9 target storefronts were equally important. The five check-marked storefronts in the whole conditions were to match the five storefronts presented in the first training session of the part conditions. The first training session ended when at least six minutes had passed and participants found all the five check-marked storefronts (indicated by hitting the event trigger poles). If participants used less than six minutes to find all the check-marked storefronts, they were encouraged to find the remaining storefronts (4) until the six-minute time limit was reached; if participants could not find all the check-marked storefronts in six minutes, the first session would not end until all of the five were found. When the first session ended, participants performed a pointing task. The second training session started when participants finished the five pointing trials.

In the second training session, participants found the remaining targeted storefronts in any order and were told to keep exploring to learn the spatial layout of this environment if there was any time left. The second training session ended when at least six minutes had passed and all the remaining storefronts were found. Participants then performed the 13 consecutive pointing task trials.

Part conditions.

The training scheme in this condition was identical to the whole conditions with the following changes: a) In the first session, the name list of the storefronts provided to participants only contained the five (out of 9) check-marked storefronts from the whole conditions. In the second session, the name list contained the remaining four target storefronts. b) In the first session, only the buildings that contained the five to-be-found storefronts were textured. The other buildings with the four storefronts to be learned in the second session were not textured (Figures 1b + d; also see video demo [https://osf.io/3bcxs]). In the second session, the untextured buildings from the first session became textured, and all but one textured building in the first session were untextured (Figure 2). The one shared textured building between session 1 and 2 encouraged participants to remember that both sessions involved the same environment. This mimicked the

two sessions training experience of the whole conditions, except that participants were not able to see the (currently) irrelevant storefronts in each of the two training sessions while still being able to perceive the full geometry of the environment. The pointing tasks in the part conditions, probing within and between phase location associations, were identical to those in the whole conditions.

Table S1: Self-report information and training behaviors across four experimental conditions.

Condition	WO	PO	WT	PT
SOD	17.00 (3.87)	17.30 (4.37)	17.25 (3.37)	17.30 (4.17)
Landmark preference	7.75 (1.89)	7.25 (1.61)	7.60 (2.15)	7.75 (1.76)
Survey preference	5.50 (2.53)	5.95 (2.31)	5.65 (1.71)	5.70 (1.52)
Total distance in training (meter)	840.76 (286.84)	765.09 (135.81)	674.97 (172.43)	647.99 (146.34)
Total time in training (minute)	14.38 (3.48)	12.86 (1.17)	13.24 (1.44)	12.52 (0.84)
Orientation change in training (degree/second)	10.32 (2.97)	10.04 (2.72)	9.14 (2.60)	10.15 (3.70)

Notes: The means and standard deviations (in parenthesis) of the self-report information and training behaviors in each experimental condition. WO – whole, opaque condition. PO – part, opaque condition. WT – whole, translucent condition. PT – part, translucent condition. SOD – sense of direction scores, possible range is from 6 to 28. Landmark preference – preference for landmark strategy, possible range is from 2 to 10. Survey preference – preference for survey strategy, possible range is from 2 to 10. Total distance in training – total traversed distance (sum of two training sessions). Total time in training – total time spent in two training sessions. Orientation change in training – the frequency participants changed their orientation in the virtual environment. No significant differences on SOD, landmark preference and survey preference were found among conditions (Fs < .18, ps > .674). Total distance was longer in the opaque conditions (WO and PO) than in the translucent conditions (WT and PT), $F(1,76) = 12.85$, $p = .001$. Total time was longer in the whole conditions (WO and WT) than in the part conditions (PO and PT), $F(1,76) = 5.88$, $p = .018$. No significant differences on orientation change across conditions (Fs < .58, ps > .45).

Table S2: Self-report information and training behaviors for participants with high SOD.

Condition	Opaque (WO and PO)	Translucent (WT and PT)
Gender	13 males, 9 females	15 males, 8 females
SOD	20.27 (2.61)	19.82 (2.73)
Landmark preference	7.95 (1.58)	7.61 (1.79)
Survey preference	6.91 (2.25)	6.21 (1.56)
Total distance in training	728.05 (118.47)	662.25 (150.35)
Total time in training	12.58 (1.22)	12.67 (0.44)
Orientation change in training	9.69 (2.70)	10.26 (3.30)

Notes: The means and standard deviations (in parenthesis) of the self-report information and training behaviors in the opaque and translucent conditions. No significant differences were found on any of the metrics (*p*s > .12). ANCOVA was used to control for all the metrics presented in this table. After controlling for these factors, participants in the translucent conditions still outperformed their counterparts in the opaque conditions in the first wayfinding task ($F(1,36) = 14.15$, $p = .001$), the third wayfinding task ($F(1,36) = 4.63$, $p = .038$), the first pointing task in the testing phase (Figure 3, testing 1) ($F(1,36) = 5.84$, $p = .021$), and the second pointing task in the testing phase(Figure 3, testing 2) ($F(1,36) = 10.13$, $p = .003$). WO – whole, opaque condition. PO – part, opaque condition. WT – whole, translucent condition. PT – part, translucent condition.

Table S3: Correlations between SOD and performance across opaque and translucent conditions.

Condition	Opaque (WO and PO)	Translucent (WT and PT)
SOD and 1st Wayfinding	-.103	-.529***
SOD and 3rd Wayfinding	-.231	-.354*
SOD and Penetrable	-.255	-.382*
SOD and Pointing_Training	-.254	-.501*
SOD and Pointing_Testing1	-.253	-.521**
SOD and Pointing_Testing2	-.146	-.619***

Notes: The correlation coefficients between SOD and performance across the opaque and translucent conditions* $p < .05$, ** $p < .01$, *** $p < .001$. WO – whole, opaque condition. PO – part, opaque condition. WT – whole, translucent condition. PT – part, translucent condition.

Table S4: Partial Correlation between SOD and performance after gender effect was controlled for.

Condition	WO	PO	WT	PT
SOD and wayfinding	-.184	-.165	-.562*	-.492*
SOD and pointing	-.014	-.030	-.523*	-.630**

Notes: The partial correlation coefficients between SOD and performance when gender effect was controlled for across experimental conditions. SOD and wayfinding – the partial correlation coefficient between SOD and the performance of the first wayfinding task. SOD and pointing – the partial correlation coefficient between SOD and the performance of the second pointing task in the testing phase. * $p < .05$, ** $p < .01$. WO – whole, opaque condition. PO – part, opaque condition. WT – whole, translucent condition. PT – part, translucent condition.

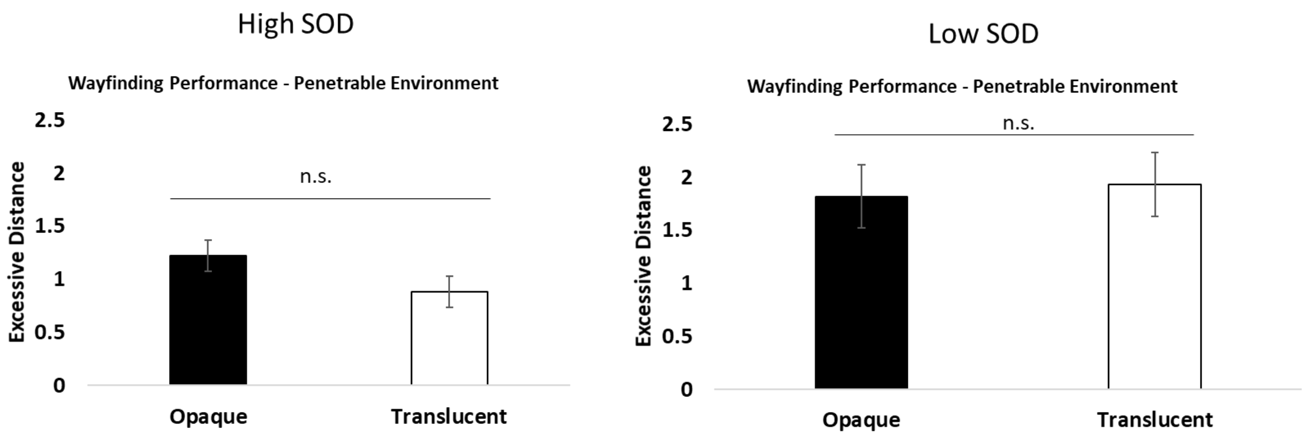

Figure S1. Wayfinding performance for high and low SOD groups in the penetrable environment. Opaque conditions were a combination of WO and PO conditions. Translucent conditions were a combination of WT and PT conditions. Error bars are ± 1 SEM estimated from data within conditions. n.s. non significant.

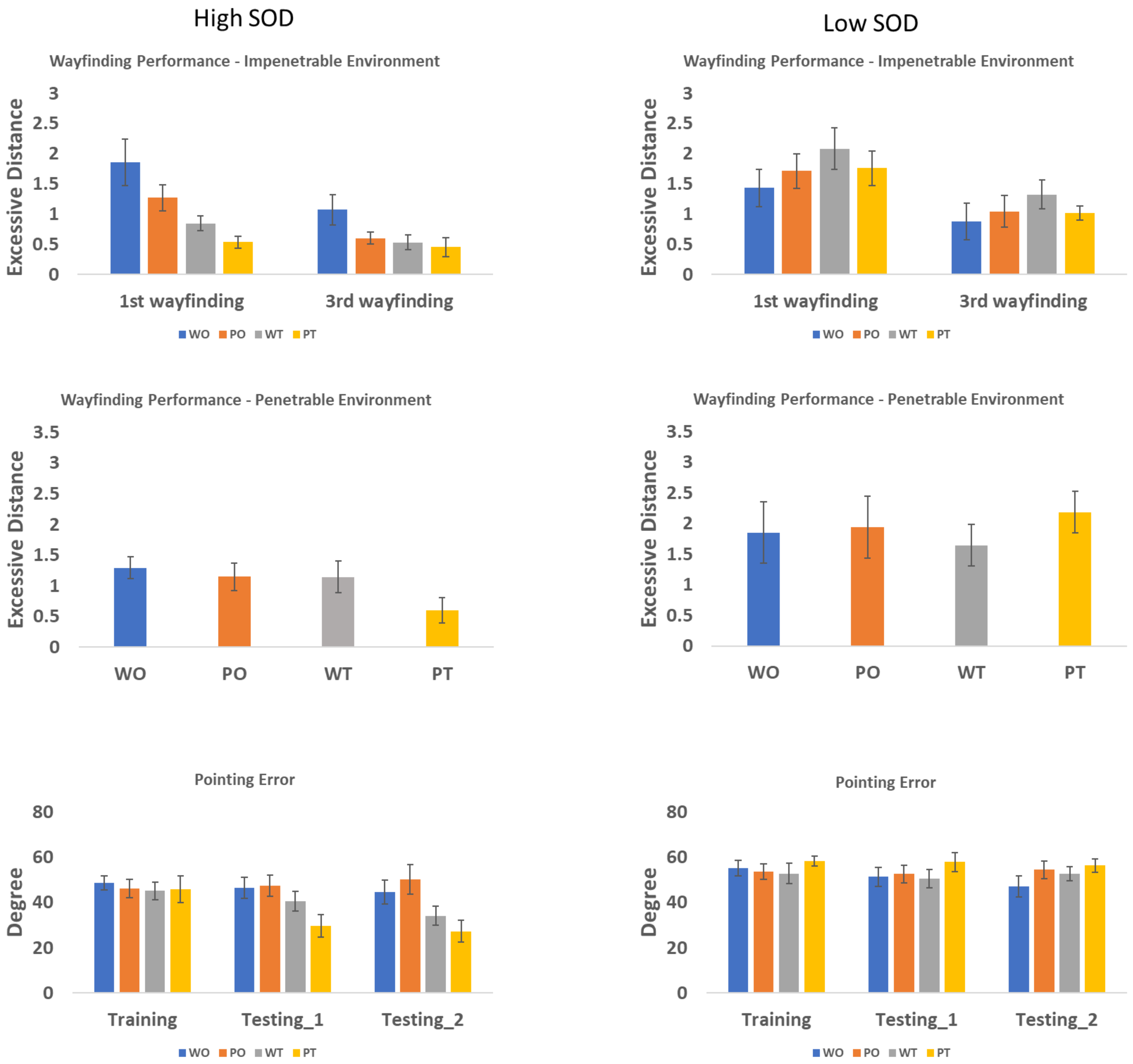

Figure S2. Wayfinding and pointing task performance for high and low SOD groups, separated by experimental condition. WO – whole, opaque condition. PO – part, opaque condition. WT – whole, translucent condition. PT – part, translucent condition.

Appendix

1. Do you think you have a good sense of direction?

1 (not at all) 2 3 4 5 (very good)

2. Are you considered by your family or friends to have a good sense of direction?

1 (not at all) 2 3 4 5 (very much)

3. Think about the way you orient yourself in different environments around you. Would you describe yourself as a person:

a. who orients him/herself by remembering routes connecting one place to

another?

1 (not at all) 2 3 4 5 (very much)

b. who orients him/herself by looking for well-known landmarks?

1 (not at all) 2 3 4 5 (very much)

c. who tries to create a mental map of the environment?

1 (not at all) 2 3 4 5 (very much)

4. Think of an unfamiliar city. Write the name . . . .

Now try to classify your representation of the city:

a. survey representation, that is a map-like representation

1 (not at all) 2 3 4 5 (very much)

b. route representation, based on memorizing routes

1 (not at all) 2 3 4 5 (very much)

c. landmark-centered representation, based on memorizing single salient landmarks (such as monuments, buildings, crossroads, etc.)

1 (not at all) 2 3 4 5 (very much)

5. When you are in a natural, open environment (mountains, seaside, country), do you naturally individuate cardinal points, that is where north, south, east, and west are?

1 (not at all) 2 3 4 5 (very much)

6. When you are in your city do you naturally individuate cardinal points, that is do you find easily where north, south, east, and west are?

1 (not at all) 2 3 4 5 (very much)

7. Someone is describing for you the route to reach an unfamiliar place. Do you prefer:

a. to make an image of the route?

1 (not at all) 2 3 4 5 (very much)

b. to remember the description verbally?

1 (not at all) 2 3 4 5 (very much)

8. In a complex building (store, museum) do you think spontaneously and easily about your direction in relation to the general structure of the building and the external environment?

1 (not at all) 2 3 4 5 (very much)

9. When you are inside a building can you easily visualize what there is outside the building in the direction you are looking?

1 (not at all) 2 3 4 5 (very much)

10. When you are in an open space and you are required to indicate a compass direction (north-south-east-west), do you:

a. point immediately?

b. need to think before pointing?

c. have difficulty?

11. You are in a complex building (many doors, stairs, corridors) and you have to indicate where the entrance is, do you:

a. point immediately?

b. need to think before pointing?

c. have difficulty?
